# Exploring the barriers and facilitators to the acceptability of donor human milk in eastern Uganda – a qualitative study

**DOI:** 10.1186/s13006-020-00272-1

**Published:** 2020-04-17

**Authors:** Sarah Magowan, Kathy Burgoine, Collin Ogara, James Ditai, Melissa Gladstone

**Affiliations:** 1 Department of International Child Health, Liverpool School of Tropical Medicine, Pembroke Place, Liverpool, UK; 2 Neonatal Unit, Department of Paediatrics and Child Health, Mbale Regional Referral Hospital, Mbale, Uganda; 3 Sanyu Africa Research Institute, Mbale, Uganda; 4 Busitema University Faculty of Health Sciences, Mbale, Uganda; 5 Department of Women and Children’s Health, Institute of Translational Medicine, University of Liverpool, Alder Hey Children’s NHS Foundation Trust, Eaton Road, Liverpool, UK

**Keywords:** Donor breast milk, Donor human milk, Neonate, Africa, Low income, Infant feeding

## Abstract

**Background:**

Human milk is the best nutrition for all infants. When the mother’s own milk is not available, the World Health Organization recommends the use of donated human milk and milk banking for neonates born prematurely or with medical problems.

Donor human milk is rarely available in low-resource settings where both the rates of preterm birth and neonatal mortality are highest. The potential to reduce neonatal mortality through use of donated human milk is one that is yet to be fully explored in the African setting. For the introduction of any new health intervention to be successful, determining the barriers and facilitators to its acceptability is a vital first step. There are limited studies on this in sub-Saharan Africa.

**Methods:**

This qualitative study used focus group discussions and in-depth interviews to explore the potential barriers and facilitators to utilizing donated human milk for neonates in a hospital setting in eastern Uganda from the perspectives of caregivers (parents, grandparents) and healthcare workers.

**Results:**

Six focus group discussions involving 28 caregivers were conducted in a hospital setting in eastern Uganda. Four in-depth interviews were then also held with healthcare staff. Lack of knowledge of donated human milk emerged with discussants, and the barriers relating to transmission of infection (HIV) and poor hygiene. Common reasons which facilitated its acceptability were; a general knowledge and recognition that human milk is better than formula milk and a strong belief by caregivers in healthcare workers providing knowledgeable and safe care. Healthcare workers were supportive of introducing donor human milk but perceived a need for community and hospital education programs to enable this to be facilitated and scaled up.

**Conclusions:**

This study shows that donor human milk can be acceptable to the caregivers of vulnerable babies in hospital settings in Uganda. Lack of awareness of donor human milk, its benefits and the methods of screening, acquisition and storage of donor milk are all barriers that could be addressed through improved education. This study advocates for national policies and programs that build capacity for effective and sustainable donor milk banking.

## Background

Human breast milk is the best source of nutrition for all infants. It is vital for infants’ growth, development and health and the World Health Organization (WHO) recommends exclusive breastfeeding for 6 months, with supplemental breastfeeding for 2 years or more [1]. For preterm and low birthweight (LBW) infants (below 2500 g), the use of breast milk becomes even more important [2]. In these high-risk infants, evidence from systematic reviews in high-income settings, shows that compared to formula milk, human breast milk reduces the risk of developing sepsis and necrotizing enterocolitis, two life-threatening diseases [3–8]. It has also been shown to reduce the incidence of retinopathy, neurodevelopmental impairment, childhood obesity and diabetes [9].

Preterm birth, maternal illness, maternal death, delay in milk production, insufficient breastmilk supply and abandonment, mean that globally up to 40% of babies in neonatal units lack access to their own mother’s breast milk [10, 11]. For these vulnerable infants, the WHO recommends donated breast milk, not formula, as the next best feeding option [1, 12]. Further, WHO recommends that if donor human milk (DHM) is needed, then it should be safely provided through a human milk bank. Human milk banking is the process by which donor breast milk is collected, screened, processed and stored thus providing a source of human breast milk for infants who would otherwise not receive it [13]. Guidelines from high income countries such as the United States recommend that lactating women who have breast milk surplus to the needs of their own infant can be invited to donate after screening for HIV-1, HIV-2, human T-cell leukemia virus 1 and 2, hepatitis B, hepatitis C and syphilis [13]. In milk banks in high-income settings, pasteurization reduces the risk of infection further. Although pasteurization also reduces the anti-infective properties and nutritional value of the breast milk, many studies have demonstrated both short and long-term benefits of donor breast milk over formula milk in preterm and LBW infants in high-income settings [3, 8, 14, 15]. In Brazil, a middle-income country, the introduction of milk banks into their newborn health policy saw neonatal mortality drop by almost three-quarters [16]. In resource-limited settings, formula milk is rarely affordable, safe or sustainable, and often either cow’s milk or poorly prepared formula milk are used when breast milk is insufficient. Donor Human Milk is therefore likely to have a larger impact on vulnerable infants in these settings. Unfortunately, donor breast milk is currently rarely available in these settings.

Of the estimated 5.9 million under-5 deaths globally in 2015, over one million of these deaths were due to complications of prematurity, thus making preterm birth complications the leading cause of under-5 deaths [17, 18]. Although the majority of preterm babies are born in low-income settings, many of these preterm deaths are still preventable as approximately 80% of preterm births occur between 32 and 37 weeks of gestation [19, 20]. Even in resource-limited settings without access to intensive care facilities, the majority of these moderate to late preterm infants can survive if simple interventions including kangaroo care and feeding support are introduced [21]. Therefore, optimal breastfeeding practices should be encouraged wherever possible and suitable for preterm infants. When breastfeeding is not feasible mothers should be supported in lactation, expression and administration of their own breast milk to their infant. For neonates with no or limited access to their mother’s own milk, efforts are needed to improve access to DHM from a human milk bank. Although DHM and milk banks are widely used in high-income settings, in the regions with the highest neonatal mortalities and the greatest number of vulnerable infants, such as sub-Saharan Africa, few human milk banks exist.

Uganda is a low-income country in East Africa with a population of 42.9 million people. In Uganda, despite advances in maternal and child health, the Neonatal Mortality Rate has not changed over two decades, remaining high at 28/1000 live births [22]. A national report from 2008 suggested that complications of prematurity accounted for 25% of these neonatal deaths [23]. It is estimated that 13.6% of births are preterm, ranking Uganda 29th highest in the world for preterm births. The United Nations (UN) seeks to reduce global neonatal mortality to 12 deaths per 1000 live births by 2030 [24].

There is an urgency to develop the availability of DHM in low-resource settings where the neonatal mortality is highest. However, for the introduction of any new health intervention to be successful, determining its acceptability is a vital first step. Only four studies have been undertaken on the acceptance of DHM in Africa including Ethiopia, Nigeria and South Africa [25–28]. No studies have been conducted in Uganda. In this study, we aim to determine the barriers and facilitators to the acceptability of donated human milk for vulnerable infants in this low-income setting from the perspective of caregivers (mothers, fathers and grandparents) as well as healthcare workers.

## Methods

This study was conducted in Mbale Regional Referral Hospital (MRRH), a public hospital that serves a population of about 4.5 million people in eastern Uganda. MRRH serves over 14 districts and their lower level health facilities. MRRH has nearly 10,000 deliveries a year and also receives neonatal referrals from surrounding health facilities. In addition, the high rate of home births means that many sick and preterm neonates are brought directly from home [22]. MRRH has a dedicated neonatal unit (NNU) that admits over 200 neonates a month, about half of whom require feeding support due to prematurity or perinatal asphyxia. Such neonates are fed either by nasogastric tube or spoon as appropriate using expressed breast milk (EBM) from their own mother. When the mother’s own EBM is limited or unavailable, the majority of mothers admitted to the NNU cannot afford formula milk. Therefore, the only options often available are currently either cow’s milk or fresh donor milk from another mother, screened for HIV, hepatitis and syphilis.

### Participants

We obtained qualitative data on the perceptions of the current barriers and facilitators towards DHM and ways to support breast milk donation and banking in the future through focus group discussions (FGDs) and in-depth interviews (IDIs). We conducted six FGDs with a mixed group of caregivers. Participants for FGDs were purposively selected. Any caregiver (mother, grandmother (“jaja”) or father) of any neonate currently, or recently, admitted in the NNU was eligible to take part. There were no specific exclusion criteria. Mixed groups were used, as we found it difficult to recruit enough men to have a group on their own. We also conducted IDIs with four of the eight healthcare workers (HCWs) from the NNU.

On a daily basis over the period of a few weeks, all potential participants meeting the inclusion criteria were identified by ward staff and research assistants. Most participants were recruited from current in-patients in the NNU and some were recruited from the weekly neonatal follow up clinic of recently discharged patients. Participants were invited to join a FGD if they could speak and understand Luganda, Lumasaaba or English, the main languages spoken locally. Each one of those potential participants was then approached by the research assistant and was provided with verbal information as well as an information leaflet (written information). Convenience sampling was used to recruit IDI participants from members of the NNU staff. All participants were required to give written consent with those who were illiterate giving a thumb print to sign their consent form. There was no remuneration for involvement.

### Data collection

IDIs and FGDs were conducted between May and June 2016. The FGDs were facilitated by two research assistants in Luganda or Lumasaaba. The HCW IDIs were conducted in English by the principal investigator. The lead research assistant was a social scientist with experience in conducting qualitative research and is fluent in Luganda, Lumasaaba and English (RN) and a note taker supported her (ST). A topic guide was used as an aide memoire to ensure that key areas were explored in each group (Additional file. Topic guide for focus group discussions on donor human milk). The topic guide was revised as data collection progressed to allow exploration of emerging issues and to enable the research assistant to probe better in areas which were felt to be important. A demographic data collection sheet was also used to gather basic information about participants. This included their age, level of education, number of children and whether they were presently breastfeeding. Name badges with a letter were provided to identify participants in the FGDs for the associated field notes to help with transcription. The IDIs were audio-recorded by the same research assistant who conducted the FGDs (RN), then translated where applicable and transcribed independently (CO or SM).

The translated IDIs were reviewed by the in-depth interviewers and compared with notes taken during the in-depth interviews to ensure accurate capturing of the data. The transcripts were imported into NVivo 10 for ease of managing the data. The translated transcripts were analyzed using an inductive thematic analysis approach by CO and SM. Familiarization with the transcripts began as each was translated. Transcripts were read several times and codes developed first through an inductive approach where any themes emerging from the data were highlighted by the coders. Following this inductive approach, the coders then went back to the topic guide and used a deductive approach to consider how the codes could be placed into themes and subthemes which related to the original topic guides. We aimed to increase credibility (the confidence that can be placed on the truth of the research findings) and trustworthiness by the use of triangulation in that we utilized different methodologies for gathering data (FGDs and IDIs) as well as different types of participants (caregivers and health care workers). We also used “member check” by feeding back to the health workers and responsive feedback on our results [29]. Furthermore, the two coders separately coded to begin with and then reviewed the coding framework prior to making a decision with a final coding framework.

## Results

There were 28 participants in the FGDs, with four to five members per group (Table 1). Only one group included a father and two out of four included only mothers. Four IDIs were conducted with HCWs (one clinical officer and three staff nurses). We provide our results thematically for both caregiver focus group discussions and in-depth interviews of health workers combined. Seven main themes arose from the analysis including; existing practices, current perceptions, fear of transmission of disease, processes of acquisition and storage of milk, external influences, transparency and health education. The barriers and facilitators are summarized in Table 2.

**Table 1:** Composition of focus group discussions with caregivers

Group	Participants	Mean age in years (range)	Mean years of schooling (range)
FGD 1	4 Mothers 1 Grandmother	29.4 (16–59)	8 (5–11)
FGD 2	3 Mothers 2 Grandmothers	37.8 (21–65)	8 (0–11)
FGD 3	4 Mothers	25.2 (21–32)	7 (3–14)
FGD 4	2 Mothers 1 Father 1 Grandmother	28.8 (18–45)	6.3 (0–11)
FGD 5	5 Mothers	27 (23–30)	10 (7–11)
FGD 6	3 Mothers 2 Grandmothers	35.2 (20–51)	8 (0–11)
All groups	28	30.6	8.9

**Table 2:** Summary of main barriers and facilitators to donor human milk

Major theme	Barriers	Facilitators
Existing practices and perceptions	Old assumptions of what is ok to provide such as cow’s milk, glucose and water	Benefits of breast milk acknowledged and some prior experience of DBM.
Fear of transmission of disease	Potential of HIV transmission	Robust, confidential HIV testing Recipient assured only negative donors permitted
	Misconception of non-communicable disease transmission	Appropriate maternal and community education
Processes of acquisition and storage of milk	Perception of poor hygiene	Ensure good hygiene practice throughout donation process
External influences	Husband as the final decision maker	Education of wider community using media and established community health resources
Transparency and health education	Negative rumors circulating in the community	Transparency and reassurance Trust in healthcare workers
	Fear of being blamed for poor outcome in recipient’s baby	Anonymous donation Appropriate education and reassurance of donor and recipient

### Existing practices and perceptions of feeding when milk is scarce

Groups of caregivers responded unanimously that water and glucose are given when no breast milk is available for newborns. Cow’s milk would typically be given if there was no milk within a few days; *“When the kid is one day, you can boil water, you cool it a bit then add glucose, and you can give like 3 spoons after 30 minutes meanwhile you wait for the breastmilk to come. At least when the kid is like 3-4 days when there is no milk completely, then you can now give cow’s milk, you boil it well. You dilute it and give to the kid” FGD 2.*

Similar responses were also given by HCWs. One HCW believed that water and glucose should be utilized in the absence of breastmilk; “Even us health workers, that first day when there is no milk, for us we have a tendency of saying there is no breast milk and we give baby water, you boil some little water, put sugar, then give this baby” HCW 4. The other HCWs acknowledge that the use of water and sugar or cow’s milk are common and suggested that mothers often lie to HCWs about what they are doing; “When there is no milk they either give cow’s milk or they give water and sugar”, “when you notice what they are doing they start lying to you” HCW 2.

The benefits of human milk over other forms of feeding were acknowledged in all FGDs and IDIs. Formula milk was mentioned in two groups, however due to its expense, was not perceived to be a common option for feeding and a contributing factor to mothers choosing to give cow’s milk. Formula milk was also mentioned by the HCWs, but again only rarely when families could afford.

The practice of someone, usually a relative, breastfeeding the baby (wet nursing) was mentioned by each group but described as something that happened in the past, before the era of HIV. *“Formally, in case the mother dies, they could just get the baby and give to another mother to breastfeed” FGD 6. “These days, things are not good, AIDS has come, so you can’t risk to breast feed your relative’s child because the mother has died, no, no it can’t happen” FGD 2.* One HCW mentioned recently seeing the practice both in the hospital and also on national television. “I have ever seen it on NTV (Ugandan TV channel). The old women were breastfeeding their grandchildren because the mothers were going to look for food” HCW 3. “I have seen a grandmother breastfeeding her grandchild. This was a young mother and she didn’t have enough milk” HCW 3.

It was clear that there was some prior understanding of the concept of DHM amongst the caregivers. One caregiver introduced the concept of donating breast milk before it had been raised by the facilitator: *“The best way is when the mother has no milk, in case you’re having someone who is looking after you or nearby and looks healthy, has no HIV and is also breastfeeding, she can press the breast in a small cup and you can use any means to give the child either using a spoon or a bottle” FGD1.*

### Fear of transmission of disease

There was fear of infectious and non-infectious transmission of diseases through breast milk. This included HIV but also non-communicable diseases including cancer, epilepsy, asthma and sickle cell disease. Fear of HIV being transmitted to their baby, either inadvertently or intentionally, was the main barrier to accepting DHM. There was the additional concern that people living with HIV may look healthy and so no one could be trusted to have “safe milk”: *“HIV virus is a lot among the people, so there is no safe person, you can say this person is healthy but the one you say is healthy is the one who is very sick” FGD 1*. Anxiety over HIV testing before donating was mentioned by two groups as a reason why some might refuse to donate. Two groups also mentioned their concerns about blame for transmitting disease (HIV) and that they might even be unfairly blamed for a bad outcome in a baby receiving their milk; *“I have sympathy to give but the child may fall sick and they say that the one who has killed our baby. It means she had a lot of infections, so I can’t, no, it can’t happen”* FGD 6. HIV was the main barrier identified by HCWs as well. One HCW commented that because HIV testing is part of routine antenatal care it may not be a problem.

The HCWs all acknowledged that the mothers would be concerned about HIV transmission; “They (the mothers) can be worried thinking the milk might be having some other infection” HCW 3. “We have told them the dangers of HIV during pregnancy, during labour and after via breastfeeding, so they know” HCW 4.

Several participants were concerned that non-communicable diseases of many types, could be transmitted through milk; *“I may be having breast cancer or blood cancer now I get my milk and give it but myself I know that I have cancer but I give saying that let it also get my disease” FGD 6. This included such conditions as; “Epilepsy, asthma*. *.*. *, these type of diseases are complicated, so I think when you breastfeed the baby with such kind of milk at times you may get such kind of complication transferred from the mother’s milk to the baby and eventually it also affects the baby in the future” FGD 4.* One HCW also remarked that caregivers might think non-communicable diseases (NCDs) could be transmitted through DHM or the baby could even change skin color, and would need educating accordingly: *“If a mzungu* (white person) *gave an African milk, that baby will not become a white, it will only get the food value out of that milk*. *.*. *if that mother had a familial disease like hypertension, diabetes, it will not be passed on through breast milk to your baby, your baby is only getting the good part of it” HCW 4.*

### Processes of acquisition, pasteurization and storage of DHM

When the process of screening, pasteurisation and milk banking was explained by the facilitators as a method of acquiring DHM the mothers were much more accepting. One mother felt that milk banking would be a good idea; *“So, for me I say like this, if they say that they are saving the lives of those children through donated milk, they should make that milk ready and store like they do blood” FGD 2*. Another mother said that if milk was acquired like blood it would be more acceptable to her because it would be more hygienic; *“The packed one, you just close your eyes and you give” FGD 6.*

Perceived hygiene of the donors and the hygiene of the donating process emerged as important influences. Mothers were particularly concerned about the lack of cleanliness of the process of acquisition of the DHM. This included the mother not being clean when she expressed the milk; *“She (the donating mother) is all over dirty”, “You don’t know how her hands looks, sometimes they express and touch dirty things and drop in the cup and that milk now contains germs” FGD 3.*

Some mothers still stated they would only accept donated breast milk if it was given by direct breastfeeding (wet nursing) because they thought it would be more hygienic; *“This milk, you can put in the cup and the fly falls there” FGD 2.* One participant even suggested that cow’s milk was more hygienically obtained than human milk, *“The hygiene is not good, meaning I will not accept such kind of condition that’s why people have always preferred that milk from the cows” FGD 4.*

### External influences (cultural/familial/health worker)

HCW were strongly influential in caregivers’ decision to accept DHM. They were perceived as knowledgeable, trustworthy and the best people to educate mothers about DHM. Participants in five FGDs remarked that they would accept DHM because of their confidence in HCWs to give safe milk: *“I don’t know where it came from but in case it’s the health worker who has given it to me, I accept because she knows where it came from” FGD 6.*

The power of husbands and HCWs in influencing decision making emerged as extremely important. Husbands were described as the final decision makers by several participants, with wives having to follow his decision: *“I need first to ask my husband, if he accepts then I accept” FGD 3, “If he refuses, then I have to leave it (using DHM) because they can chase me away from the home” FGD 6.*

When the influence of religious leaders and community elders on DHM was explored, participants did not describe these leaders as being influential, particularly as they did not have the relevant knowledge on this subject. Despite this, cultural beliefs still held a strong influence with three groups describing a local belief that giving breast milk from another mother is poisonous; *R: “So people in our place if they hear that you’re going to express milk and give to another person, they know that you’re going to kill that person”. I: “that you’re going to kill the child?” R: “yes, that you’re going to kill”; I: “so they were saying that all those who express, children die?” R: “yes” FGD 4.*

The influence of community rumors was suggested as a reason by healthcare workers for parents refusing medical care*; “They might think maybe there is some poison. So sometimes when you are giving you have to be clear because they might be thinking that you want to kill their babies” HCW 3.*

### Importance of transparency and health education

HCWs said that as DHM would be a new intervention, caregivers may treat it with suspicion due to lack of experience or knowledge of its use, but this could be overcome with education. *“This kind of thing, donation of milk, is rare here, it is rare so when they start it* (in NNU)*, they (the caregivers) may first imagine ‘Why? Why is it now that they want to do this?” HCW3. “It is something which has never been happening so it is a new thing but I believe when you teach, in time people will get used” HCW2.*

Caregivers expressed the need for sensitization; “We need information on what you’re supposed to do before you donate” FGD 2. The education should come from the health workers; “That information we should get from the hospital or in the health centre or the village health teams” FGD 6. Many caregivers felt that the community and not just the mothers should be sensitized; “I need them to sensitize all the people to be aware of it, myself, the entire village and the local chairperson” FGD 6.

Providing caregivers with appropriate information and explaining clearly the milk had come from a mother who had tested negative for HIV, was described as a means of increasing recipients’ acceptance by each HCW. *“I think we need to make things plain to them and say, ‘ok we’ve got milk from another mother, we’ve tested and here are the results, is it ok with you?’ But if we are doing it like behind doors, I think it will bring some bit of mistrust” HCW 1. “When you talk to someone, I believe they can understand. Because otherwise they can say ‘Donated milk, maybe they are bringing from chimpanzee, they are bringing from those animals, it’s the one they are bringing to feed their baby’” HCW 2.*

Interestingly, three HCWs thought it was important for mothers to know exactly who the donating mother was. However, one felt it was not important if the recipient could be shown evidence that the donors were not infected with infections like HIV, hepatitis or syphilis. In her opinion, the important issue was proving the donors were healthy: *“What we have to assure them is we get milk from voluntary people, healthy people, if someone is sick of any condition like*. *.*. *HIV we first test them, see that someone is healthy” HCW 4.*

Each HCW shared the belief that with education and time, DHM could become an acceptable practice on the ward and seen as part of normal care for certain babies. *“I think it can work. It will be the first time it will be part of treatment, like you give ampicillin, you give these fluids, you tell them there is milk and you first explain to her” HCW 4.*

Educating the wider community was felt to be important for DHM to become an acceptable practice. The two most common areas identified for education were the safety of DHM and its benefits compared with cow’s milk or formula. The use of media, especially radio, to sensitize the community about any new intervention was felt to be important. Engaging the already established village health teams (VHT) to educate and raise awareness of DHM was suggested as an important means of introducing the concept of DHM and increasing acceptance by the community. The VHTs were regarded as being respected by the community.

## Discussion

This study has provided in-depth qualitative information on the facilitators and barriers to providing DHM in a small hospital setting in Uganda. We used qualitative methodology which included focus group discussions and in-depth interviews in order to enable us to dive deeper into the issues which need addressing. The focus groups enabled participants to voice ideas and to listen to the ideas of others and further develop new thoughts and were also a way of enabling more information in a short time. The IDIs were particularly useful for persons who were experts. In addition, the IDIs enabled us to have a more private discussion individually with each of the HCWs without them influencing each other.

We demonstrated how caregivers are understandably most concerned about the risk of infection. Caregivers clearly stated that DHM would be acceptable with the assurance that donors had been adequately screened for HIV and other transmissible diseases alongside appropriate hygiene measures during the donation and safe pasteurization. Similar studies in South Africa, Nigeria and Ethiopia, also identified the caregiver’s primary concern relating to DHM was the possibility of HIV transmission [25, 27, 28, 30]. The perception that non-communicable diseases could be transmitted through DHM was also reported in the Ethiopian study, where mother’s voiced concerns over transmission of cancer and acne. This study highlighted additional concerns about transmission of epilepsy, asthma and sickle cell disease. The implication of this finding highlights the importance of robust education and reassurance of caregivers, their spouses and the wider community. This could be effectively achieved through health education in the community, antenatal clinics and other facility based educational programs.

Caregivers explained how much they trust the HCWs, who advise the correct and most beneficial treatment for their infants, influenced their acceptance of DHM. Participants displayed an unanticipated level of confidence and trust in HCWs. In South Africa, although many mothers voiced similar opinions, there were also expressions of mistrust in healthcare providers that limited their acceptance [25]. Our results demonstrate that for the introduction of DHM or any other novel intervention, a trusting relationship between the patient and the HCW is vital. Maintenance of these trusting relationships is likely to be best done through robust training and education of HCWs in order that they can play a strong role in advocacy.

**Fig. 1:**
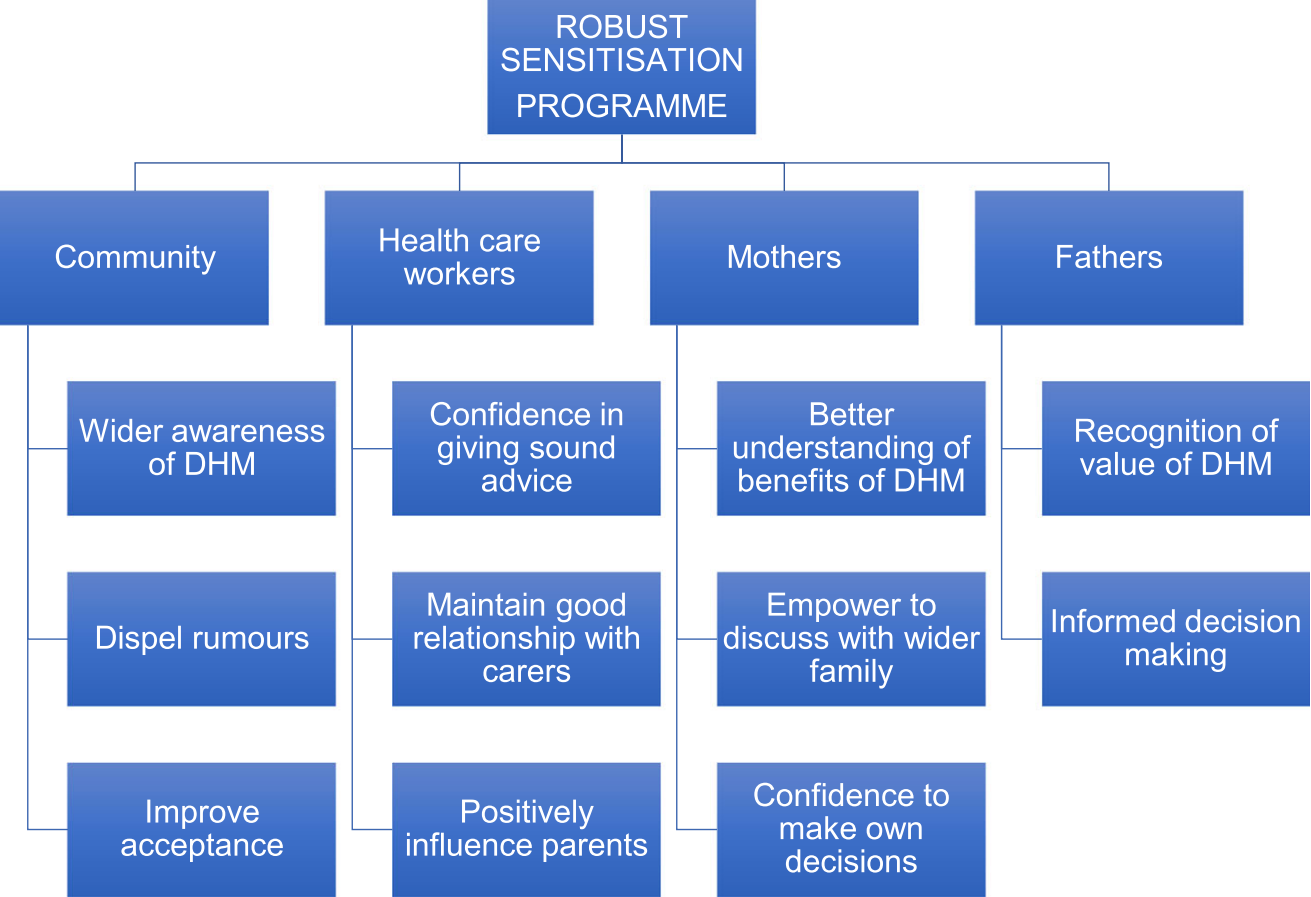
Role of education in improving donor human milk acceptance

Education and improved awareness of the beneficial role of DHM will be vital in generating acceptance and allowing it to become part of routine neonatal care. In Ethiopia, acceptance of DHM was over five times more likely among mothers who had previously heard of it than those who hadn’t. The need for education was also a dominant theme in our qualitative work but in addition, the final decision of the mother to accept DHM was heavily influenced by her husband and family. Mothers cannot be educated in isolation and it is clear that any education program would need to involve the whole family. Similarly, in Nigeria 80% of women require spousal consent prior to donating milk [28]. In order to move forward, we must therefore address the need for better education and increased awareness in community settings, during antenatal care and in the neonatal unit. Although not mentioned by any of our participants, it is likely that government support and approval would be a vital factor in the success of breastmilk donation and banking. We propose a robust sensitization program (Fig. 1) which would support the acceptability of donated human milk in the future. As shown in Fig. 1, such a program should be diverse to ensure the sensitization of all those supporting the mother. This should include the father and the community, so that they all have the necessary education to support her appropriately to make her own decisions. Whilst negative perceptions exist and lack of knowledge on the safety and true benefit of DHM exist, the implementation of DHM in any setting will be challenging. Healthcare workers have the ability to positively influence parents and are reportedly the most trusted individuals in healthcare decisions. It is pertinent therefore that the HCWs need to be educated about DHM and milk banking and be given training in informed consent to maximize this intervention.

The processes of acquisition and storage of DHM were found to be incredibly important to the caregivers in our study, particularly when relating to hygiene. Interestingly, this was not mentioned by the HCWs. A study in Ethiopia also identified this as important, although not the study conducted in South Africa [25, 27]. Strict infection control protocols are encouraged within the NNU in Mbale, which may explain why participants shared this concern. Healthcare workers may have assumed that good hygiene practices would be used during donation and so had not perceived it as a barrier.

Our study, as well as previous South African and Nigerian studies identified the need for caregivers to know who the breast milk donor was [25, 26, 28]. Caregivers in Ethiopia were keen only to have DHM if it was from a blood relation. Although this might be feasible on small scale, large scale donation and milk banking would not support this, but this might need to be addressed at least in the information provided for caregivers.

In many aspects of life in Uganda, religious leaders and community elders have strong influence on decision making for communities, however we did not find examples of this within our research. Most participants in this study were Christian, although a few Muslim participants were involved and surprisingly, were in favor of using donor milk. Milk kinship is a belief Muslims hold that if a mother donates her milk, the recipient becomes like her own child and is seen to be a sibling to her own children. This has significant implications for marriage, as “milk siblings” are forbidden to marry, and therefore makes the concept of milk banking unpopular in predominantly Muslim settings [31, 32]. In the Ethiopian study, Muslim participants voiced a preference for formula or cow’s milk over DHM. Uganda is a mostly Christian country and therefore the findings of this study may not be generalizable to neighboring countries with higher proportions of Muslims. Muslim law does however allow the use of donated human milk if strict conditions are met, for example if it is pooled from the milk of at least three donors, therefore further discussion and education may improve acceptability in certain settings [33]. This may need to be explored further in future studies.

### Study limitations

Our study was small with only six mixed FGDs and four IDIs and only limited demographic information about participants. Participants were recruited solely from a hospital setting where opinions may differ widely from those held in the community and more rural settings. As all participants had delivered in hospital, pre-existing trust and acceptance of health care may be more evident with participants more open to new interventions such as DHM. Husbands often have the final say in decision making, but unfortunately very few men were present in the NNU and therefore we had very limited views from fathers. Conducting FGDs in the community may produce very different responses.

The respondents in this study were mainly Christian so the views of other religions are not well represented. In addition, views of policy makers were not collected, these will be vital for the future implementation of human milk banking in Uganda. Any future studies should determine the perspectives of these additional groups.

## Conclusions

This study has demonstrated that DHM can be acceptable to the caregivers of vulnerable babies in a hospital setting in Uganda where neonatal mortality is high and where every effort must be made to reduce it. Lack of awareness of DHM, its benefits and the methods of screening, acquisition and storage of donor milk are all barriers that could be addressed through improved education at various levels. Donor Human Milk has the potential to have a huge impact on neonatal mortality and morbidity in low-resource settings like Uganda. Therefore, campaigns are needed to raise awareness about DHM whilst simultaneously establishing national policies and programs that build capacity for effective and sustainable donor milk banking.

## Supplementary information

**Additional file 1.** Topic guide for focus group discussions on donor human milk.

## Data Availability

The datasets used during the current study are available from the corresponding author on reasonable request.

## Abbreviations

DHM: Donor human milk
EBM: Expressed breast milk
FGD: Focus group discussion
HCW: Healthcare worker
HIV: Human immunodeficiency virus
IDI: In-depth interview
LBW: Low birthweight
MRRH: Mbale Regional Referral Hospital
NCD: Non-communicable disease
NNU: Neonatal Unit
WHO: World Health Organization
