# Supplementary material for: Exploring the barriers and facilitators to the acceptability of donor human milk in eastern Uganda – a qualitative study
Source: Int Breastfeed J. 2020 Apr 17;15:28. doi: 10.1186/s13006-020-00272-1 (PMC7165402; doi:10.1186/s13006-020-00272-1)
Supplement: Supplementary file 1 — Additional file 1. Topic guide for focus group discussions on donor human milk. [file 13006_2020_272_MOESM1_ESM.docx]

Topic guide for focus group discussions on donor human milk

| **Tell the group that sometimes mothers may have difficulty feeding their babies because they might not be able to produce enough milk or the mother might be unwell after birth.** | |
| --- | --- |
| **Domain** | **Topic and Probes** |
| **CURRENT PRACTICE WHEN MOTHER DOES NOT HAVE MILK** | - In this situation (where the mother cannot produce enough milk) what could be given to feed the baby?   ***Probe: response from each mother***   - Tell me more about the choices   ***Probe: each mother who has given choices should respond to the reason for the choice***   - Tell me a story – does anyone know of anyone in the community who had a mother from the community feed their baby?   ***Each mother should try and tell her story and ask these probes:***   - How did that work? - What were the barriers? - What made it work? - Tell me more about that? - Has it always happened this way? |
| **THE INFLUENCES ON HOW MOTHERS FEED** | - Who influences how you feed your baby and what to do if there are problems? (lack of milk or baby not taking milk)   ***Probe: is it the spouse/in-law/mother/siblings etc***   - Who would be able to help mothers in feeding their baby in the home/where they live?   ***Probe: for responses from each participant – in-law/sibling/mother/friends/TBA?***   - What might wise and influential people say about using another mothers milk? (Village health team, health workers, community leaders, cultural leaders) - What might mothers think about using another mother’s milk? - What would fathers/uncles/grandfathers think?   ***Probe: Each participant to share their opinion*** |
| **PERCEPTIONS OF DONOR MILK** | **Tell the group that sometimes we have to give blood to newborn babies that has been donated from other people. In some countries they give milk to babies that has been donated from other mothers who have extra milk.**   - Tell me what you think about milk from other mothers being given to another mother’s baby?   ***Probe for reasons***   - Have you heard of such a practice?   ***Probe: where have they heard? From who have they heard?***   - Do you know of examples?   ***Probe: for each participant who has commented ask for further details – did it work? Were there problems?***   - If you could not produce milk, what do you think you would do if you were asked to give milk from other mothers to your baby?   ***Probe: ask for a response from each participant***   - What do you think your friends would do? - Who might need to give you permission? Or help you to decide? Tell me more about this.   ***Probe: ask about in-laws, spouse, mother, father, siblings etc?***   - Who decides whether this might be OK in the community?   ***Probe: ask about those outside of the family - clan leaders, village leaders etc***   - What do you think might be things that would cause this (giving other mother’s milk) to be a problem?   ***Probe: ask each mother who has responded to explain*** |
| **PAST PRACTICES** | - Tell me if you know if this has happened in the past? (other mothers donating milk for others babies)   ***Probe: ask each participant***   - Do you have stories that you know about other mothers giving milk? Can you share.   ***Probe: ask each participant who has responded yes*** |
| **PERCEPTIONS AND BARRIERS TO DONATION** | - Would you be willing to donate your milk to others?   ***Probe: ask each mother and probe for explanation to response***   - Would you allow your spouse/sibling/daughter in law donate milk to others?   ***Probe: ask each respondent for explanation for their response***   - Do you think there are differences between donated milk and donated blood? And tell me what you think these differences might be?   ***Probe: ask each respondent for explanation***   - What information would mothers need if they were going to give their baby donated milk?   ***Probe: ask about infection (HIV, hepatitis)***   - What information would mothers need if they were going to donate their milk? - Who else should be given this information?   ***Probe: ask about spouse/in-laws?***   - Who should give this information?   ***Probe: midwives at ANC, health care worker, village health team, school teachers, church/mosque?*** |
